# Supplementary material for: Frequency comb generation via synchronous pumped χ(3) resonator on thin-film lithium niobate
Source: Nat Commun. 2024 May 9;15:3921. doi: 10.1038/s41467-024-48222-3 (PMC11082247; doi:10.1038/s41467-024-48222-3)
Supplement: Supplementary file 1 — Supplementary Information [file 41467_2024_48222_MOESM1_ESM.pdf]

Supplementary Note 1 | Effect of annealing on dispersion

The resonator device operates in a near-zero normal dispersion regime. To achieve this dispersion in conjunction with high quality factor, the resonator devices are annealed after fabrication. The annealing process slightly changes the bulk material dispersion, which in turn moves the waveguide dispersion from the anomalous regime into the normal regime. Using index data collected on thin-film LN wafers before and after annealing, we simulate and plot the group velocity dispersion $\beta_2 = \frac{\partial^2 k}{\partial \omega^2}$ and the dispersion operator $D = \sum_{n=2,3,\cdots} \frac{\beta_n(\omega_0)}{n!} (\omega - \omega_0)^n$ (Figure S1).

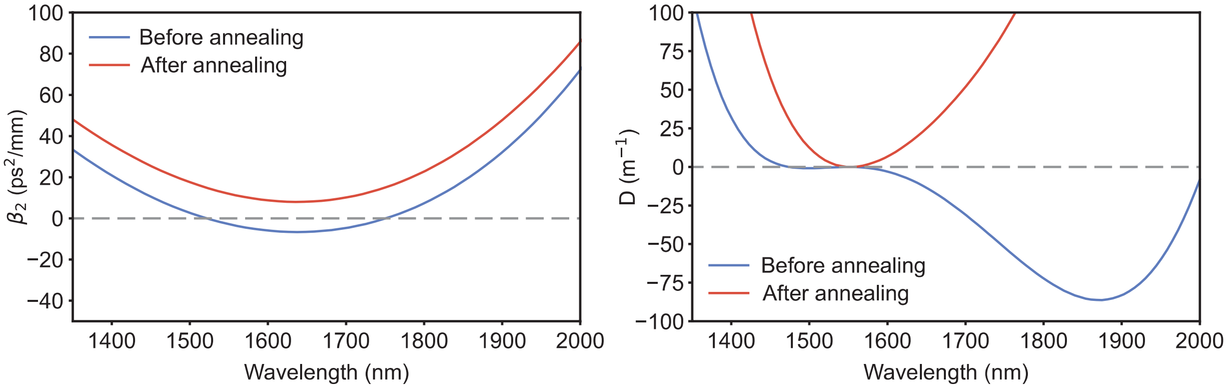

Figure S1 | Group velocity dispersion (left) and dispersion operator (right) curves of resonator waveguide geometry before and after high temperature furnace annealing. The annealing process pulls the dispersion from the anomalous into normal regime.

Supplementary Note 2 | Extended measurement layout

The full measurement scheme for the pulse-pumped case is given in Figure S2a. A tunable laser is fiber coupled to the pulse generator chip. The light is polarization controlled using fiber polarization paddles to ensure coupling to the TE optical mode and passed through a series of on-chip amplitude and phase modulators. The modulators are driven using GSG (ground-signal-ground) probes and an off-chip signal generator, amplifier, pre-amplifier, variable attenuator, and phase shifter. Pulses are coupled off-chip and sent to a pulse amplifier before being coupled to the resonator chip. The length of fiber between the two LN chips, including fiber in the EDFA and the polarization control, is set to 59 m, which corresponds to maximum compression of the outgoing pulse train. The final spectrum from the resonator is measured on an optical spectrum analyzer.

For testing the resonator under continuous-wave conditions, the pulse generator chip is bypassed completely and only one optical setup is used. The tunable laser is amplified and sent

directly into the resonator chip (Figure S2b). An optical circulator is used to allow monitoring of the spectrum in both the forward and backward directions. The spectrum is collected in the backward direction for analysis.

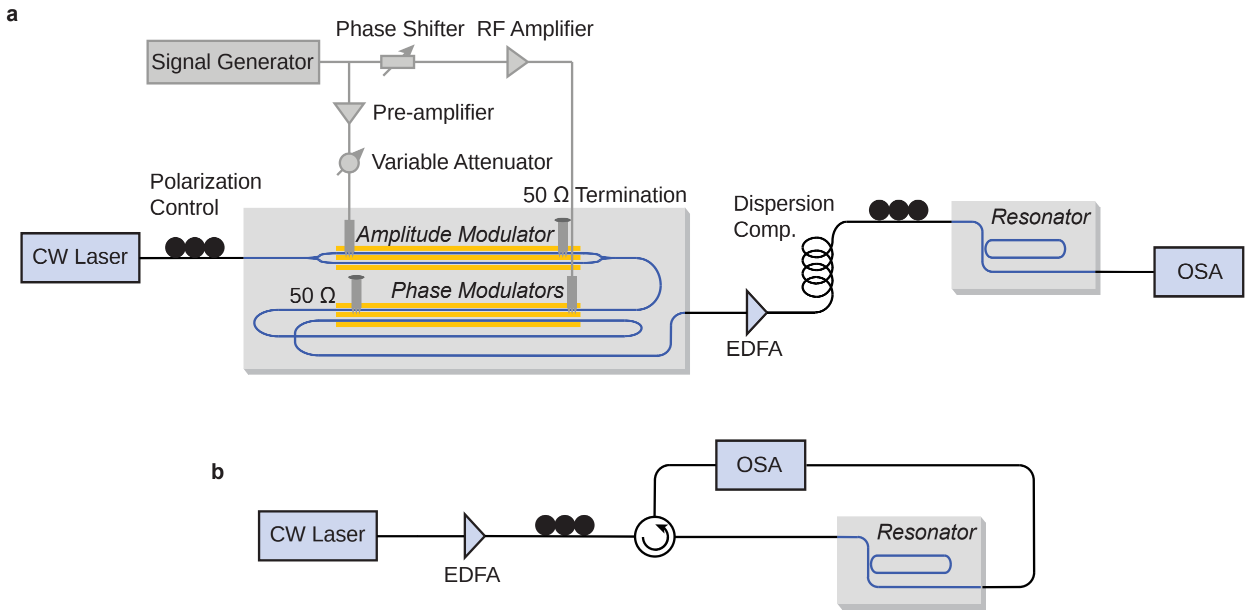

Figure S2 | Experimental setup. a, Setup schematic for pulse generation and driving across two LN photonic chips; **b,** Setup for testing spectrum under CW pumping. A circulator is used to monitor the spectrum in the backward direction. EDFA: erbium doped fiber amplifier; OSA: optical spectrum analyzer.

Supplementary Note 3 | Repetition rate detuning

The effect of repetition rate detuning on the spectrum is studied from 0 to 3 MHz detuning at 1 MHz steps with ~6 mW average on-chip power on the resonator chip. The effect on the spectrum is visible at 2 MHz detuning and becomes significant at 3 MHz detuning, at which the stability of the spectrum is also greatly sacrificed (Figure S3). We believe that the effects of repetition rate detuning will be more stringent at higher optical powers, where the spectrum bandwidth is larger. We note that the hump in the OSA floor around 1530 nm is due to unfiltered ASE noise from the EDFA source used between the setups.

We note that the characteristics of the spectra in Figure S3 and Figure S4 differ slightly from the spectrum in the main body text. This is posited to be due to aging of the resonator sample

over long time periods between measurements, which cannot be completely recovered through reannealing of the sample.

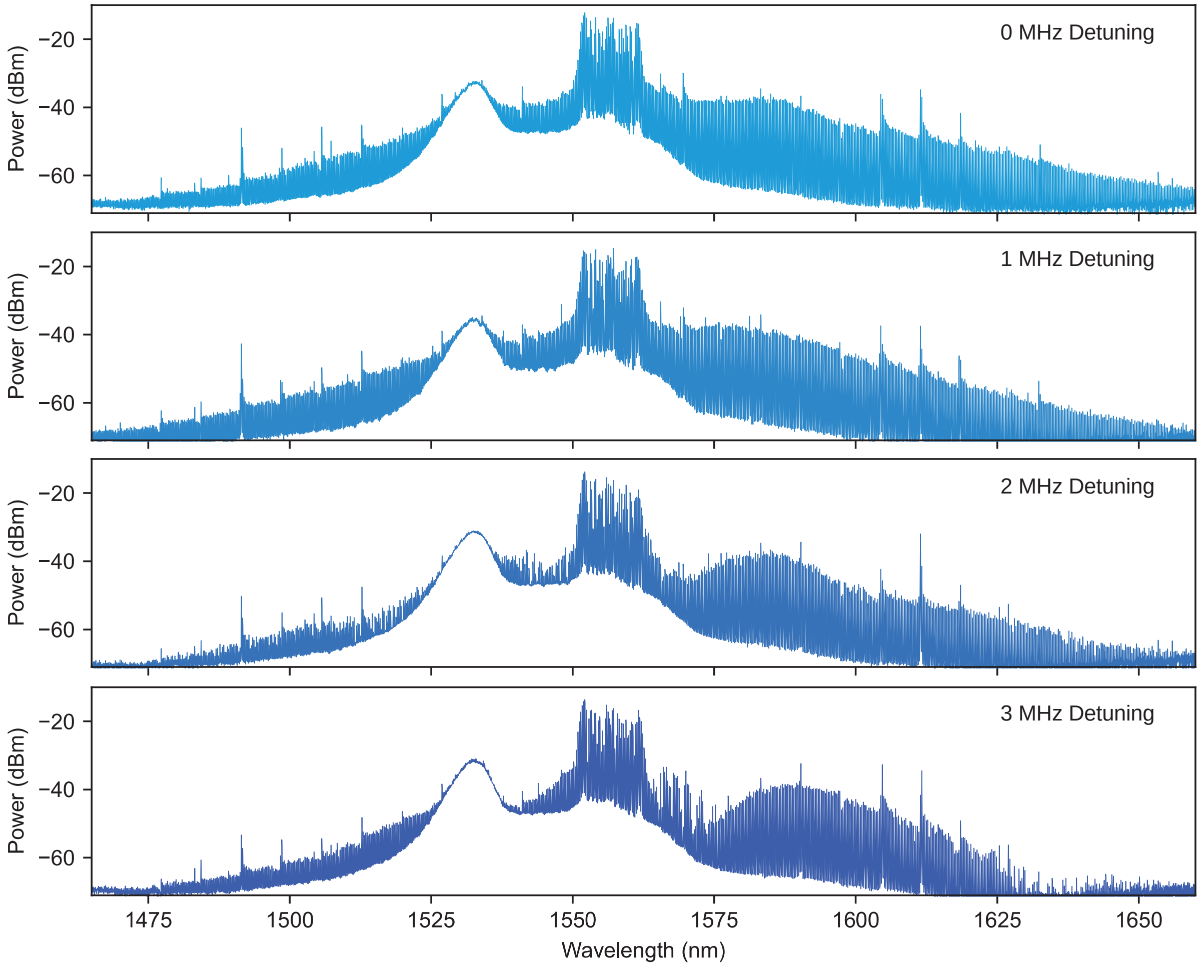

Figure S3 | Optical spectrum collected at 0-3 MHz RF detuning, pumping with 6 mW average on-chip optical power.

Supplementary Note 4 | Optical power

We test the broadening behavior of our system by tuning the EDFA gain to change the on-chip power of the resonator chip, which allows for control of the power without any effect of the optical dispersion between the chips. The output spectrum from the resonator is measured for a range of average on-chip optical powers from 1.0 to 5.0 mW (corresponding to 64 mW to 320 mW of peak power and 36 fJ to 0.18 pJ of pulse energy). The corresponding spectra are given in Figure S4.

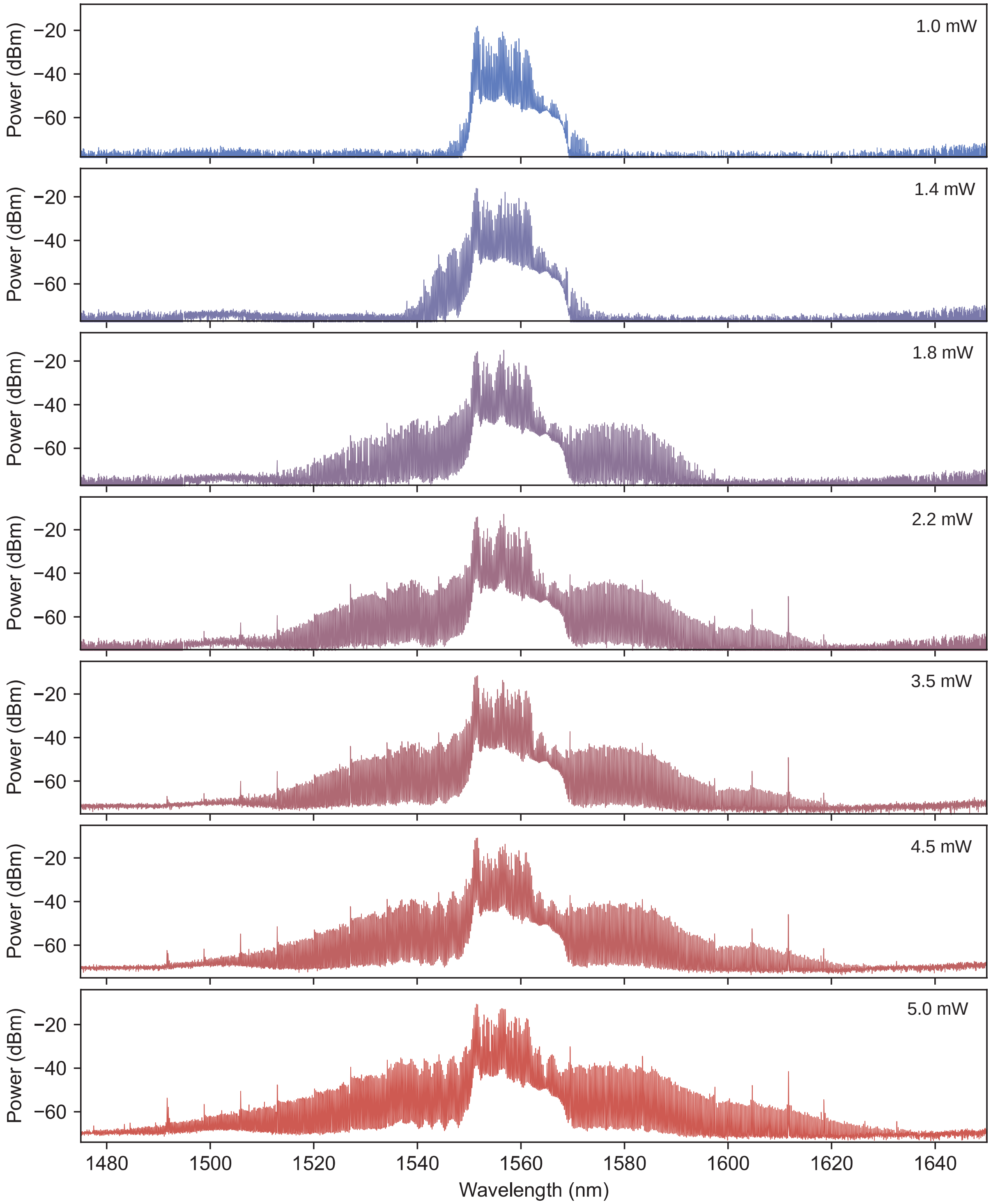

Figure S4 | Generated optical spectra for average on-chip powers ranging from 1.0 to 5.0 mW. The power is adjusted by tuning the current on the EDFA. As expected, we see broader comb spectrum for increasing optical power.
